# Microcirculation vs. Mitochondria—What to Target?

**DOI:** 10.3389/fmed.2020.00416

**Published:** 2020-08-05

**Authors:** Tamara Merz, Nicole Denoix, Markus Huber-Lang, Mervyn Singer, Peter Radermacher, Oscar McCook

**Affiliations:** ^1^Institute for Anesthesiological Pathophysiology and Process Engineering, Ulm University Medical Center, Ulm, Germany; ^2^Clinic for Psychosomatic Medicine and Psychotherapy, Ulm University Medical Center, Ulm, Germany; ^3^Institute for Clinical and Experimental Trauma-Immunology, University Hospital of Ulm, Ulm, Germany; ^4^Bloomsbury Institute for Intensive Care Medicine, University College London, London, United Kingdom

**Keywords:** circulatory shock, oxidative stress, hypoxia, organ failure, inflammation

## Abstract

Circulatory shock is associated with marked disturbances of the macro- and microcirculation and flow heterogeneities. Furthermore, a lack of tissue adenosine trisphosphate (ATP) and mitochondrial dysfunction are directly associated with organ failure and poor patient outcome. While it remains unclear if microcirculation-targeted resuscitation strategies can even abolish shock-induced flow heterogeneity, mitochondrial dysfunction and subsequently diminished ATP production could still lead to organ dysfunction and failure even if microcirculatory function is restored or maintained. Preserved mitochondrial function is clearly associated with better patient outcome. This review elucidates the role of the microcirculation and mitochondria during circulatory shock and patient management and will give a viewpoint on the advantages and disadvantages of tailoring resuscitation to microvascular or mitochondrial targets.

## Introduction

Shock can be defined as the “*imbalance between oxygen supply and requirements*” (1). This imbalance can be due to “*inadequate O*_2_ *transport*” resulting from “*hypovolemia*,” “*cardiogenic factors*” (e.g., myocardial infarction), “*obstruction*” (e.g., pulmonary embolism), and/or “*distributive shock*” (e.g., septic shock), which is characterized by “*decreased systemic vascular resistance and altered oxygen extraction*” (1). Hence, cellular hypoxia is central to shock pathophysiology. Current resuscitation strategies mainly address macrocirculatory targets to restore appropriate tissue perfusion, but achieving these targets does not necessarily result in improved microcirculatory perfusion, since there may be a marked dissociation between the former and the latter (2–4).

**Figure 1:**
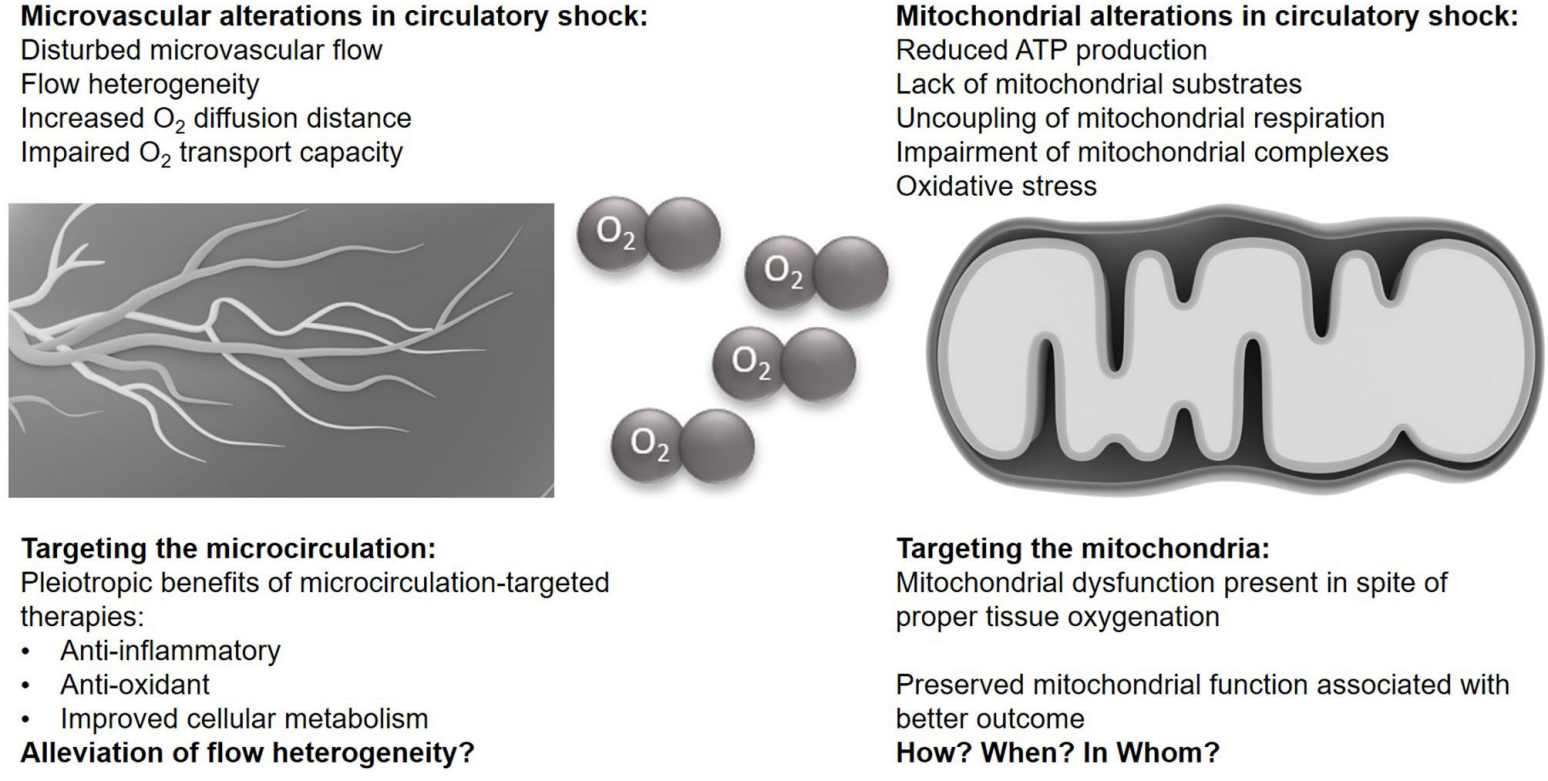
The microvasculature and mitochondria in circulatory shock. Illustrations of the microcirculation and the mitochondrium are taken from the Library of Science and Medical Illustrations (somersault18:24, https://creativecommons.org/licenses/by-nc-sa/4.0/).

It is beyond any doubt that circulatory shock, no matter whether it is septic (5) or traumatic-hemorrhagic (i.e., hypovolemic) (6), is associated with marked disturbances of the microcirculation. Moreover, it is well-established that survivors present with improved markers of microcirculatory perfusion (7, 8). Nevertheless, it remains a matter of debate as to whether this is an epiphenomenon or a causal relationship, in other words, whether “*recruiting the microcirculation*” (9) or “*microvascular resuscitation*” (10) is the “*magic bullet*” that will improve survival after circulatory shock. This question still raises fairly equivocal viewpoints (9, 11, 12). Trezciak et al. (13) failed to demonstrate any relationship between changes in microcirculatory markers and the severity of organ failure. Furthermore, in resuscitated patients with septic shock, a direct relationship was noted between eventual outcome and skeletal muscle adenosine trisphosphate (ATP) (14), suggesting a perhaps more important role for cellular metabolic capacity compared to microcirculatory O_2_ supply, at least in sepsis. This review covers the respective roles of the microcirculation and mitochondria in circulatory shock (summarized in Figure 1), with a particular focus on traumatic-hemorrhagic vs. septic shock. It addresses the question as to whether altered mitochondrial respiration and subsequently diminished ATP production could still lead to organ dysfunction and failure, despite adequate tissue oxygenation, even if microcirculatory function is restored or maintained (15, 16).

## Evidence for Impaired Microcirculatory Perfusion During Shock

There is numerous experimental and clinical evidence for impaired microcirculatory perfusion during shock, no matter whether the origin is sepsis or trauma-hemorrhage (i.e., hypovolemic). In experimental animal models, impaired microcirculatory perfusion has been demonstrated using various techniques in the heart, kidney, liver, gut, and brain, even after resuscitation had restored the macrocirculatory hemodynamics (17–22).

The disturbance of the microcirculation is characterized by a markedly enhanced heterogeneity of blood flow comprising obstructed vessels, vessels with stagnant or intermittently on/off flow related to vasoconstriction, and vessels with an increased blood flow velocity (2). All these effects, together with an increased O_2_ diffusion distance (e.g., due to tissue edema) and/or reduced systemic O_2_ transport capacity (e.g., due to hemodilution) will result in impaired tissue O_2_ availability (4). In fact, in the intestinal mucosa, increased O_2_ extraction was directly related to the degree of these microcirculatory disturbances (23). Finally, the increased heterogeneity of the intestinal mucosal microcirculation, characterized by a substantial proportion of non-perfused capillaries, coincided with increased regional venous lactate/pyruvate ratios, a well-established marker for cellular dysoxia, and tissue acidosis (17).

This experimental evidence is supported by clinical observations. In a seminal study in stabilized septic patients, De Backer et al. (5) demonstrated the presence of marked microcirculatory disturbances characterized by a decreased density of perfused small vessels, and a large number of either non-perfused or only intermittently perfused vessels. While the proportion of perfused capillaries was not related to systemic macro-hemodynamics, it was significantly lower in non-survivors. This group (7, 24) and others have subsequently confirmed these findings in patients with sepsis (8, 25, 26) and following trauma-hemorrhage (6).

## Does “Microvascular Resuscitation” Help?

Given the importance of an impaired microcirculation, “*microvascular resuscitation*” (10) or “*recruiting the microcirculation*” (9) has been advocated. Strikingly, few clinical studies have been published to date that integrate a microcirculation-targeted resuscitation into the study protocol. One, by Boerma et al. (27), found no outcome benefit from targeting the sublingual microcirculation with nitroglycerin. Whether such an approach may provide a “*magic bullet*” to improve survival after circulatory shock has generated conflicting views (9, 11, 12). This is in part due to the ongoing lack of readily accessible data from bedside techniques that assess the microcirculation, notwithstanding the conclusions of a recent consensus conference (28). Moreover, despite the undoubtedly existing impairment of microcirculatory perfusion in shock, the subsequent conclusion that tissue O_2_ availability is reduced to a meaningful degree has been questioned, at least in resuscitated septic shock: in one study, not only was skeletal tissue PO_2_ not reduced in such patients, but—in sharp contrast to patients with cardiac pump failure—was even higher than a healthy control group (29).

**Figure 2:**
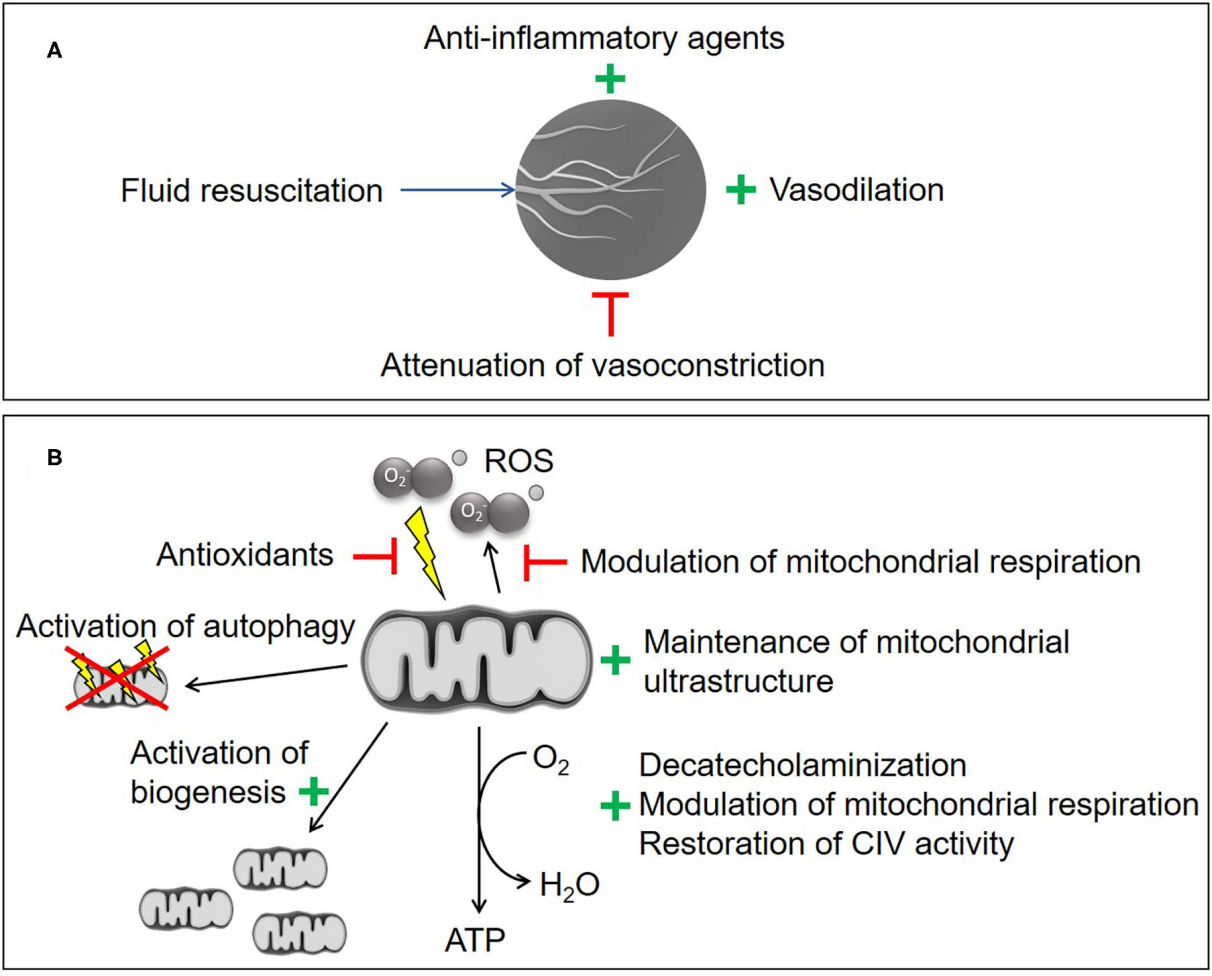
Experimental therapeutic strategies. **(A)** Microvascular recruitment. **(B)** Mitochondrial protection and stimulation. CIV, cytochrome c oxidase; ROS, reactive oxygen species. Illustrations of the microcirculation and the mitochondrium are taken from the Library of Science and Medical Illustrations (somersault18:24, https://creativecommons.org/licenses/by-nc-sa/4.0/) .

Any recruitment maneuver targeting the microcirculation will comprise two aspects, i.e., the “*(re-)opening*” of the capillary network (e.g., using fluid resuscitation, ino-dilation and restriction of vasoconstriction) and the subsequent attenuation of flow heterogeneity (summarized in Figure 2A) (2). Based on this rationale, fluid resuscitation (30–34), dobutamine (35), levosimendan (36), milrinone (37), nitric oxide (NO) donors (38–40), and prostacyclin (PGI_2_) (40–42) have all been tested. In experimental settings, this approach was often successful but the majority of animal studies were only of short duration and/or did not include standard intensive care measures, which may limit transferability into clinical practice (43). Given the potential NO- or NO-derivatives induced uncoupling of mitochondrial respiration and inhibition of complex I and complex IV (14, 44–46), caution must be taken with NO-donors as a therapeutic strategy to avoid detrimental effects on the mitochondria. In longer-term, fluid-resuscitated large animal models, therapeutic interventions that attenuated shock-related cellular dysoxia (e.g., selective inhibition of the inducible NO synthase (iNOS), antioxidant infusion, therapeutic hyperoxia) revealed that any beneficial effect on the microcirculation coincided with improved parameters of inflammation, oxidative and nitrosative stress, and/or cellular metabolism (21, 47, 48). It thus remains unclear whether the beneficial effect on the microcirculation is the cause or the result of these responses. Indeed, neither selective iNOS inhibition nor infusion of the PGI_2_ analog iloprost had any effect on the measured parameters of microcirculation at all, but still resulted in improved markers of cellular dysoxia (49, 50).

To date, the results of clinical interventional studies that have integrated monitoring of microcirculatory perfusion and oxygenation have failed to show a direct relationship between the effects of the respective intervention on the measured markers of microcirculation and either mortality and/or morbidity. For example, Ospina-Tascon et al. (51) found that fluid resuscitation (infusion of at least 1,000 ml Ringer's lactate or 400 ml 4% albumin solution) within the first 24 h of diagnosis of sepsis increased the fraction of perfused small vessels, and this effect was directly related to a decrease in lactate concentration. Beyond 48 h of diagnosis, no effect was observed (51).

Dobutamine can recruit the microcirculation in septic patients but, again, its effects were directly related to a decrease in lactate levels, rather than the individual macrocirculatory response (52). More recently, Hernandez et al. (53) failed to demonstrate any beneficial effect of dobutamine neither on the sublingual microcirculation nor on metabolic, hepatosplanchnic or peripheral perfusion parameters. Based on a theoretical benefit of vasodilating drugs (54–56) the use of NO releasing compounds has been investigated. Administration of nitroglycerin increased the perfusion of sublingual microvessels (57), but, as mentioned previously, continuous infusion over 24 h trended to a worse outcome in a study including 70 patients (*p* = 0.08) (27). Inhaling 40 ppm of NO over 6 h after initial resuscitation neither improved microcirculatory flow, lactate clearance, nor organ dysfunction, and no association was found between parameters of microcirculatory perfusion and organ dysfunction after initial resuscitation (13). Based on promising results with infusion of PGI_2_ (or its analogs) (58, 59), a multicenter randomized controlled trial is currently under way in patients with septic shock and “persistent microperfusion defects” (I-MICRO, NCT03788837). However, it must be underscored that PGI_2_ and its analogs have effects beyond those on the microcirculation (60), in particular with respect to cellular energy metabolism (61–65). Whether or not vasoconstrictors have deleterious effects on the microcirculation has also been recently questioned. In healthy volunteers, neither norepinephrine, phenylephrine nor vasopressin affected microcirculatory parameters prior to or after bolus injection of endotoxin, despite an immediate rise in blood pressure (66). Overall, a recent meta-analysis on the impact of vasoactive drugs on microcirculatory blood flow concluded that there is “*no robust evidence that any agent can lead to improved microvascular flow*” and that “*no study demonstrated outcome benefit*” (67).

## Evidence for Cellular Energetic Failure Resulting From Reduced Mitochondrial Respiratory Activity

Some two decades ago, the late Mitchell Fink created the term “*cytopathic hypoxia*” (68, 69). The above-detailed disturbances of the microcirculation could result in inadequate cellular O_2_ supply as a reason for decreased ATP production and subsequent hyperlactatemia. Clearly, aggravation of microvascular heterogeneity with mismatch of the local O_2_-transport/ uptake relationship could explain at least in part, why the threshold of O_2_ supply, below which hyperlactatemia occurs, is much higher during sepsis than under normal conditions (70, 71). In this context, the concept of cytopathic hypoxia (or, perhaps, more accurately, cytopathic dysoxia) attempts to reconcile the phenomenon that organ failure coincides with hardly any cell death, availability of oxygen at the cellular level, relatively minor inflammatory cell migration, and capacity of these failed organs to recover (72, 73). Cytopathic dysoxia refers to impaired ATP formation despite normal [or even supra-normal (20)] tissue PO_2_ levels. It can result from several factors, for instance, diminished delivery of key substrates (e.g., pyruvate) into the tricarboxylic acid (TCA) cycle, inhibition of various TCA cycle or (in particular) electron transport chain enzymes such as Complexes I and IV (74, 75), and uncoupling [“slipping” (76)] of the electron transport chain with a decrease in the proton gradient across the inner mitochondrial membrane resulting in production of heat rather than generation of ATP.

To date, the assessment of mitochondrial function is mostly limited to *ex vivo* methods, which might misrepresent the *in vivo* situation. A detailed discussion of available methods for *ex vivo* and *in vivo* mitochondrial measurements are beyond the scope of this paper, but have been recently compared by Bettink et al. (77), with a particular focus on the protoporphyrin IX-triplet state lifetime technique, which is applicable *in vivo* (78). However, in the light of the absolute values reported for the latter *in vivo* measurement technique, which range between 30 and 110 mmHg (77, 79), the results obtained with this method have to be interpreted with care. In contrast to these “mitochondrial” PO_2_ levels, reports of tissue PO_2_ measured with the Pd-phosphorescence quenching method typically range between 20 and 25 mmHg, with maximally 52 mmHg and even levels as low as 5 mmHg detected, which is the threshold for tissue to be considered anoxic (80). A steep O_2_ pressure gradient is needed to facilitate O_2_ diffusion from capillaries through the interstitium to cells/mitochondria. Thus, it is not surprising to see reports of intracellular PO_2_ of no more than 1–10 mmHg (81). Mitochondrial ATP supply is stable over a wide range of intracellular PO_2_, measured *ex vivo* by oxygraphy, and will only suffer if the mitochondrial PO_2_ is lower than 0.1–0.5 mmHg (81). Thus, the values reported for mitoPO_2_ of 30–110 mmHg may represent a mixture of PO_2_ values from different compartments rather than solely mitoPO_2_.

Despite these limitations in determining mitochondrial function, there is ample experimental evidence that reduced (disturbed) mitochondrial respiratory activity assumes crucial importance for shock-related organ dysfunction or failure, similar to the potential role of impaired microcirculatory perfusion and oxygenation. Several rodent studies that included resuscitation demonstrated hyperlactatemia despite unchanged or even increased tissue PO_2_, and this coincided with both reduced function and structural damage to mitochondria (74, 82–85). Resuscitated models investigating higher species (cats, swine, baboons) and characterized by a normotensive and a normo- or even hyperdynamic circulation, confirmed these findings (20, 86–89). In this context, studies simultaneously recording parameters of both microcirculation and cellular O_2_ utilization assume particular importance: LPS-challenged, fluid-resuscitated swine showed a reduced efficiency of hepatic mitochondrial respiration despite maintained liver surface laser Doppler blood flow (20). The same group however reported in a longer-term model, unchanged liver tissue mitochondrial resuscitation with a tendency toward enhanced microcirculatory blood flow (88). They undertook a meta-analysis reviewing both experimental models and clinical studies and reported variable results on mitochondrial function depending on the species, organ, and time point investigated (90). However, the available experimental literature mainly originates from young and otherwise healthy animals, which does not represent the more frequent clinical scenario of elderly patients with comorbidities, a common pitfall of experimental studies in shock research in general (43). Nevertheless, there is elegant clinical evidence that despite adequate resuscitation, reduced mitochondrial respiration is (i) present in patients after the initial management of circulatory shock, and (ii) associated with worse outcomes. In a landmark study in patients with septic shock, complex I activity was lower in non-survivors than in survivors; and corresponding tissue ATP content values mirrored this result (14). Moreover, complex I activity was inversely related to the mitochondrial antioxidant, glutathione, and directly related to nitrate/nitrite concentrations, suggesting a crucial role of oxidative and nitrosative stress in shock-related mitochondrial (dys)function. The same group subsequently demonstrated that impaired mitochondrial function coincided with a decrease in mitochondrial respiratory protein content in non-survivors. In contrast, survivors showed an early activation of mitochondrial biogenesis (91). A *post-hoc* analysis of the HYPER2S trial (92) in patients with sepsis-induced hypotension found that hyperoxia during the first 24 h of treatment increased mortality rate at day 28 (*p* = 0.054) in hyperlactatemic patients, whereas in those with lactate levels ≤ 2 mmol/L there was no effect on mortality or morbidity. Since hyperoxia increases tissue O_2_ partial pressure, even under conditions of profound reduction of O_2_ supply (93), this finding implicitly suggests that outcome of septic shock is associated with impaired cellular O_2_ utilization rather than microcirculatory O_2_ availability, possibly due to aggravated oxidative and nitrosative stress (94).

## Does “Mitochondrial Resuscitation” Help?

Multiple mitochondria-targeted therapeutic strategies have been proposed (summarized in Figure 2B) (95–98). Despite several promising experimental therapies, there are currently no clinically-approved strategies for mitochondrial protection. Metformin has been shown to be beneficial for the attenuation of mitochondrial transition pore opening (99), stimulation of mitochondrial biogenesis and reduction of mitochondrial ROS production, but has to be used with caution in shock patients due to its potentially severe side effects, i.e., renal impairment, and lactic acidosis (100).

In contrast, the glucose-lowering compound Imeglimine, though to date only tested experimentally, has none of the side effects as metformin, but inhibited mitochondrial permeability transition, improved mitochondrial function, and was associated with less acidosis (100, 101). The inhibition of the mitochondrial permeability transition pore with cyclosporine A showed promise in a pre-clinical sepsis model and a large-animal model of traumatic brain injury (102, 103). In humans, cyclosporine A did not show a benefit in cardiac arrest and acute myocardial infarct (104, 105), and it was never clinically approved for the treatment of any type of circulatory shock.

Given the above-mentioned role of oxidative and nitrosative stress, mitochondria-targeted antioxidant strategies and manipulation of shock-related excess formation of nitric oxide (NO) and/or peroxynitrite (ONOO^−^) have been suggested but so far, despite promising results even in clinically relevant, resuscitated large animal models (47, 48, 106–110), none have made their way into clinical practice. Other strategies have targeted restoration of cytochrome-c-oxidase (Complex IV) activity (111, 112), maintenance of mitochondrial inner membrane integrity (113), activation of autophagy to clear damaged mitochondria to promote biogenesis (114), direct activation of mitochondrial biogenesis (95), and modulation of mitochondrial respiration using gaseous mediators (115, 116). To date, only experimental evidence is available for all of the latter strategies. In contrast, tight blood glucose control preserved both mitochondrial function and ultrastructure, and, thereby, attenuated organ dysfunction independently of organ perfusion in a rabbit model of prolonged critical illness (85). The protective effect of this strategy was associated with better maintenance of mitochondrial activity and morphological integrity in patients (117).

In the context of mitochondrial dysfunction-induced organ failure, the “*decatecholaminization*” paradigm (118, 119) may assume particular importance. It is well-established that catecholamines, beyond their effects of hemodynamics, have profound immune- and metabolism-modulating properties (120, 121), ultimately resulting in “*metabolic stress*” (122), the degree of which is directly related to their β-adrenergic activity (123). Catecholamines inhibit cellular respiration *in vitro* in a dose-dependent manner (124, 125). In clinically relevant, resuscitated, large animal models, the degree of impaired mitochondrial respiration and, ultimately, organ dysfunction was directly related to the norepinephrine infusion rates required to achieve hemodynamic targets (55, 89). It is tempting to speculate that this finding is related to the well-known norepinephrine-related aggravation of oxidative (126, 127) and nitrosative stress: in patients, nitrite/nitrate concentrations were inversely related to both tissue complex I activity and glutathione concentrations, but directly related to norepinephrine requirements which, in turn, coincided with low complex I activities (14).

## Conclusion

Both microvascular and mitochondrial alterations are consequences of circulatory shock and associated with worse outcomes. However, none of the promising microvasculature- or mitochondrial-targeted pre-clinical therapeutic approaches have yet translated to clinical practice. The challenges of assessing microvascular and/or mitochondrial function in patients limit the current understanding in this field. Simultaneous measurements of both parameters, such as recently performed by Rutai et al. (128) in a pre-clinical study, might help to alleviate this issue. To date, it remains unclear whether any of the microcirculation therapies currently under investigation will be successful in abolishing shock-induced flow heterogeneity, rather than having beneficial effects through their anti-inflammatory and/or anti-oxidant properties. Similarly, mitochondrial dysfunction may result in organ failure despite adequate tissue perfusion and oxygenation. Therefore, a crucial question to address in the future is: how, when and in whom should we protect/support the mitochondria?

## Data Availability Statement

The original contributions presented in the study are included in the article/supplementary material, further inquiries can be directed to the corresponding author/s.

## Author Contributions

PR envisioned, drafted, and corrected the final version of the manuscript. TM drafted, created the figures, and edited the final version of the manuscript. MS contributed to the draft and edited the final version of the manuscript. ND, OM, and MH-L edited the manuscript. All authors read and approved the final version.

## Conflict of Interest

The authors declare that the research was conducted in the absence of any commercial or financial relationships that could be construed as a potential conflict of interest.
